# The UDP-glucosyltransferase multigene family in *Bombyx mori*

**DOI:** 10.1186/1471-2164-9-563

**Published:** 2008-11-27

**Authors:** Fei-Fei Huang, Chun-Li Chai, Ze Zhang, Zeng-Hu Liu, Fang-Yin Dai, Cheng Lu, Zhong-Huai Xiang

**Affiliations:** 1 The Key Sericultural Laboratory of Agricultural Ministry, Institute of Sericulture and Systems Biology, Southwest University, Chongqing 400715, PR China; 2 The Institute of Agricultural and Life Sciences, Chongqing University, Chongqing 400044, PR China

## Abstract

**Background:**

Glucosidation plays a major role in the inactivation and excretion of a great variety of both endogenous and exogenous compounds. A class of UDP-glycosyltransferases (UGTs) is involved in this process. Insect UGTs play important roles in several processes, including detoxication of substrates such as plant allelochemicals, cuticle formation, pigmentation, and olfaction. Identification and characterization of *Bombyx mori *UGT genes could provide valuable basic information for this important family and explain the detoxication mechanism and other processes in insects.

**Results:**

Taking advantage of the newly assembled genome sequence, we performed a genome-wide analysis of the candidate UGT family in the silkworm, *B. mori*. Based on UGT signature and their similarity to UGT homologs from other organisms, we identified 42 putative silkworm UGT genes. Most of them are clustered on the silkworm chromosomes, with two major clusters on chromosomes 7 and 28, respectively. The phylogenetic analysis of these identified 42 UGT protein sequences revealed five major groups. A comparison of the silkworm UGTs with homologs from other sequenced insect genomes indicated that some UGTs are silkworm-specific genes. The expression patterns of these candidate genes were investigated with known expressed sequence tags (ESTs), microarray data, and RT-PCR method. In total, 36 genes were expressed in tissues examined and showed different patterns of expression profile, indicating that these UGT genes might have different functions.

**Conclusion:**

*B. mori *possesses a largest insect UGT gene family characterized to date, including 42 genes. Phylogenetic analysis, genomic organization and expression profiles provide an overview for the silkworm UGTs and facilitate their functional studies in future.

## Background

All organisms live in an environment that contains natural and man-made potentially harmful chemicals. Extensive studies of detoxification in the vertebrate liver provide a framework to the study of detoxification mechanisms in other systems [1-3]. Several biotransformation enzymes participate in the detoxification process, they are cytochrome P450 superfamily [4,5], short chain dehydrogenase/reductase (SDR)1 family [6], glutathione S-transferases [7] and UDP-glucuronosyltransferases [2].

Biotransformation enzymes related to those found in vertebrates have also been found in insects and are likely to play equally important roles. Cytochrome P450s and glutathione S-transferases in particular have been implicated in insect resistance to pesticides [8]. UDP-glucuronosyltransferases are part of a superfamily of UDP-glycosyltransferases (UGTs, EC2.4.1.-) that are found in all living organisms, including animals, plants, bacteria and viruses, suggesting an ancient origin [9-11]. UGTs are a superfamily of enzymes that mediate the transfer of glycosyl residues from activated nucleotide sugars to acceptor molecules (aglycones), thus regulating properties of the acceptors such as their bioactivity, solubility and transport within the cell and throughout the organism. The UDP-sugar may be UDP-glucuronic acid, UDP-galactose, UDP-glucose, or UDP-xylose.

The mammalian UGTs using UDP-glucuronic acid as a glycosyl donor have attracted considerable attention in pharmaceutical and clinical research due to their central role in the detoxification of foreign chemicals such as carcinogens and hydrophobic drugs. These enzymes are located in the lumen of the endoplasmic reticulum and are membrane-bound protein. In addition to the detoxication of exogenous substrates, they are involved in a range of other physiological processes, including olfaction and metabolism of bile acids and steroids [12].

Like plant UGTs, insect UGT enzymes also use UDP-glucose rather than UDP-glucuronic acid as sugar donor [13-15]. Also, similar to the vertebrates, both endogenous and exogenous substrates are subject to glucosidation in insects. UGT activity on endogenous and exogenous compounds has been reported in a range of insect species [16]. Insect UGTs play an important role in detoxication of plant allelochemicals encountered by many insects in their diets [14]. Consequently, UGT-catalyzed biotransformation of xenobiotics has been implicated in some cases of insecticide resistance [17]. In addition, insect UGTs play important roles in several processes, including cuticle formation, pigmentation, and olfaction [18-20]. However, only limited molecular information on insect UGTs is available.

Genome sequencing projects offer a new route into understanding multigene families both within a single species and across different species. In the present study, we have used the newly assembled 9× coverage genome sequence  to characterize the UGT multigene family in silkworm. In total, the 42 putative silkworm UGTs were identified. The phylogenetic relationships among these genes and the homologs from the sequenced insect genomes were analyzed. Searching available ESTs and microarray data, we found that 36 of 42 silkworm UGTs were expressed in different tissues, suggesting that these genes are active and may have different functions. In addition, the genomic structures of silkworm UGT genes were also investigated. Our data provide the preliminary insights into evolution and functions of the silkworm UGTs. This is the first time to describe this gene family in a Lepidoptera species. The results may have important implications for the study of insect UGTs.

## Results

### The UGT family number of silkworm

**Figure 1:**
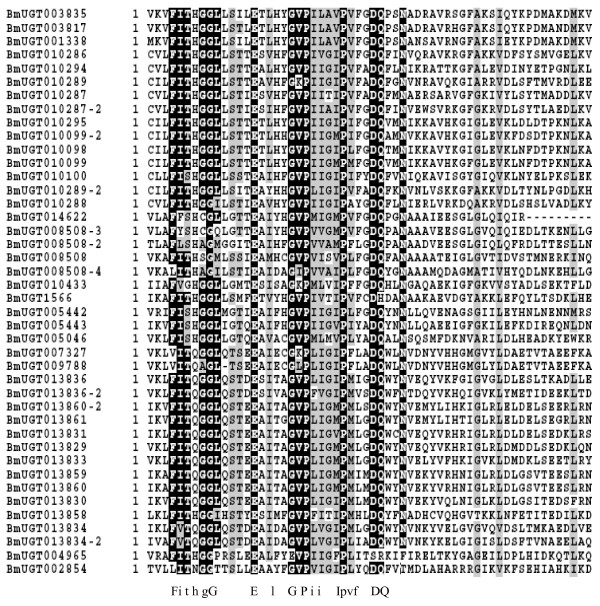
**The signature motif of silkworm 42 UGT genes**. Alignment of UGT amino acid sequences. Black and grey indicate identical and similar amino acids, respectively. Multiple sequence alignment was performed with CLUSTALW and amino-acid shading with BOXSHADE 3.21 . A consensus is indicated in the region of the UGT signature sequence. (FVA)-(LIVMF)-(TS)-(HQ)-(SGAC)-G- X(2) -(STG)-X(2)- (DE)-X(6)-P-(LIVMFA)-(LIVMFA)-X(2)-P-(LMVFIQ)-X(2)- (DE)-Q, (all amino acids that can concur at a given position are listed inside brackets; X indicted any amino acid; reviewed in [10])

A signature sequence involved in the binding of the UDP moiety of the nucleotide sugar has been identified as a characteristic of UGT sequences from a range of prokaryotic and eukaryotic organisms. To gain insight into the size of the UGT family in the silkworm, the amino acid sequence corresponding to this signature motif in insect UGTs was used to screen the predicted silkworm protein database. The reported amino acid sequences of *Drosophila melanogaste*r UGTs were also used as queries for the BLASTP searches. We ultimately identified 42 UGT genes from the silkworm genome (Table 1). The signature motifs are well conserved in the silkworm UGT genes (Figure 1). Similarly, we also identified 22 and 12 UGT genes from *Anopheles gambiae *and *Apis mellifera *genomes, respectively. It has been reported that there are 33 UGTs in the *D. melanogaster *genome. It is obvious that the *B. mori *genome contains more members of the UGT family compared with *A. gambiae*, *A. mellifera*, and *D. melanogaster*. Among all of the identified silkworm UGT sequences, the N-terminal region is more variable than the C-terminal region where the signature sequence resides.

**Table 1:** Summary of the silkworm UGTs. UN indicates the unknown chromosome locations of the UGTs.

Gene ID	Protein length	Exon	Chr.	Scaffold	Domains	EST	Probe
BmUGT014622	504	4	UN	scaffold968	UDPGT	0	sw06973
BmUGT001338	500	8	UN	nscaf1987	UDPGT	1	sw22710
							
BmUGT007327	497	4	3	nscaf2882	UDPGT	0	
BmUGT004965	496	4	25	nscaf2822	UDPGT	43	sw09395
BmUGT013836	475	4	28	nscaf3098	UDPGT	1	sw14525
BmUGT013836-2	489	4	28	nscaf3098	UDPGT	1	sw05627
BmUGT003835	510	8	24	nscaf2686	UDPGT	0	sw04632
BmUGT003817	480	8	24	nscaf2686	UDPGT	5	sw19163
							
BmUGT013858	505	4	28	nscaf3098	UDPGT	0	
BmUGT013834	475	4	28	nscaf3098	UDPGT	0	sw20757
BmUGT013834-2	514	4	28	nscaf3098	UDPGT	0	sw19970
BmUGT013833	515	4	28	nscaf3098	UDPGT	0	sw20758
BmUGT013831	512	4	28	nscaf3098	UDPGT	2	sw20803
BmUGT013830	497	4	28	nscaf3098	UDPGT	0	sw18651
BmUGT013829	514	4	28	nscaf3098	UDPGT	1	sw18729
BmUGT013859	520	4	28	nscaf3098	UDPGT	2	sw22688
BmUGT013860	521	4	28	nscaf3098	UDPGT	3	sw19839
BmUGT013860-2	510	4	28	nscaf3098	UDPGT	3	sw21445
BmUGT013861	514	4	28	nscaf3098	UDPGT	3	sw22761
BmUGT005442	519	4	8	nscaf2828	UDPGT	0	sw05898
BmUGT005443	508	4	8	nscaf2828	UDPGT	0	sw01020
BmUGT010433	499	4	12	nscaf2993	UDPGT	1	sw09899
BmUGT005046	505	4	25	nscaf2823	UDPGT	2	sw15861
BmUGT009788	497	4	2	nscaf2964	UDPGT	0	sw04199
							
BmUGT010286	505	8	7	nscaf2986	UDPGT	1	
BmUGT010287	515	8	7	nscaf2986	UDPGT	3	sw19569
							
BmUGT010287-2	499	8	7	nscaf2986	UDPGT	0	
BmUGT010288	494	8	7	nscaf2986	UDPGT	0	sw13662
BmUGT010289	499	8	7	nscaf2986	UDPGT	4	sw19370
BmUGT010289-2	502	8	7	nscaf2986	UDPGT	4	sw18102
BmUGT010100	498	8	7	nscaf2986	UDPGT	4	sw19481
							
BmUGT010294	520	8	7	nscaf2986	UDPGT	4	
							
BmUGT010295	515	7	7	nscaf2986	UDPGT	1	
							
BmUGT010099	510	8	7	nscaf2986	UDPGT	4	
							
BmUGT010099-2	504	7	7	nscaf2986	UDPGT	4	
BmUGT010098	501	7	7	nscaf2986	UDPGT	4	sw19365
							
BmUGT008508	500	4	18	nscaf2902	UDPGT	2	
							
BmUGT008508-2	497	4	18	nscaf2902	UDPGT	0	
							
BmUGT008508-3	508	4	18	nscaf2902	UDPGT	0	
							
BmUGT008508-4	494	4	18	nscaf2902	UDPGT	1	
BmUGT002854	505	7	10	nscaf2575	UDPGT	0	sw06704
							
BmUGT1566	-	2	UN	nscaf1566	partialUDPGT	0	

### Phylogeny of the silkworm UGT superfamily

**Figure 2:**
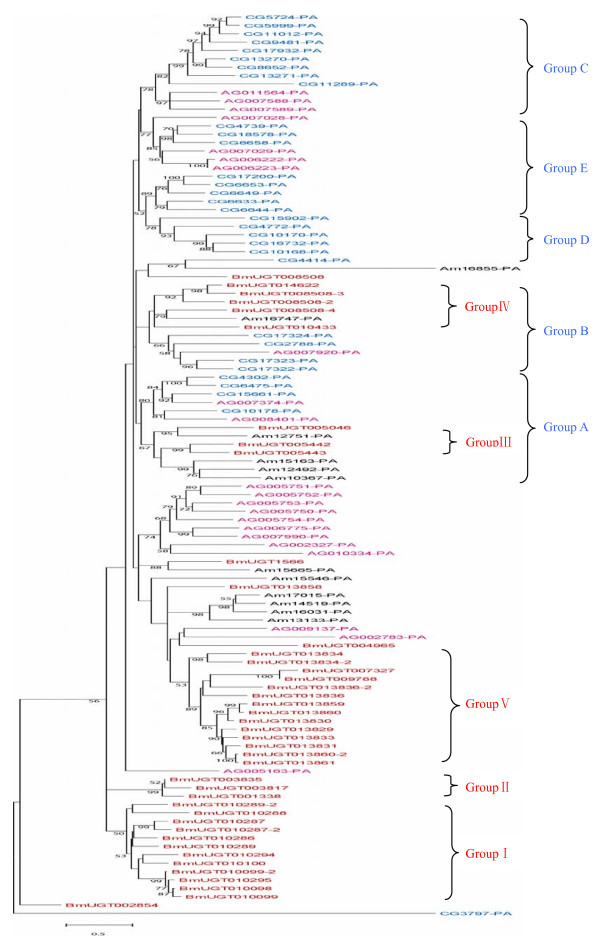
**Neighbour-joining tree of *B. mori*, *A. gambiae*, *A. mellifera *and *D. melanogaster *UGTs**. Phylogentic tree was reconstructed with MEGA 4 program. Genetic distance was computed based on Jones-Taylor-Thornton model and gaps were deleted with pairwise deletion method. Bootstrap values (1000 replicates) lower than 50% were omitted. *B. mori *(Bm), *A. gambiae *(Ag), *A. mellifera *(Am) and *D. melanogaster *(CG) UGTs were presented by red, pink, black, and blue, respectively.

**Figure 3:**
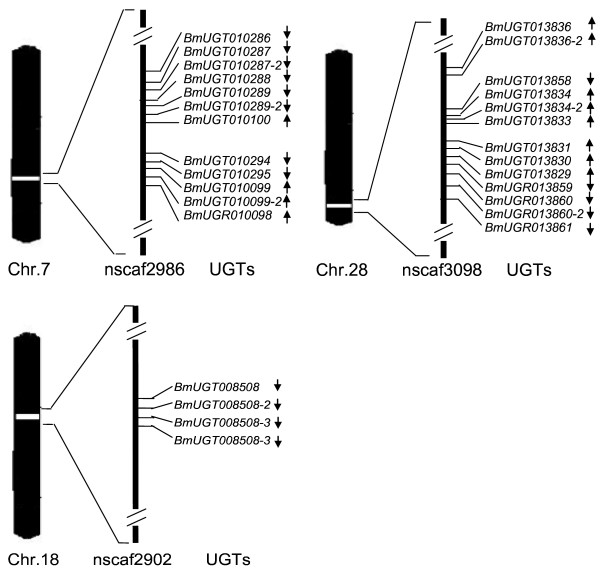
**The positions of *B. mori *UGT genes on chromosome**. The black arrow indicates the transcription direction of UGTs. Only the chromosomes that have more than 2 UGTs were shown.

**Figure 4:**
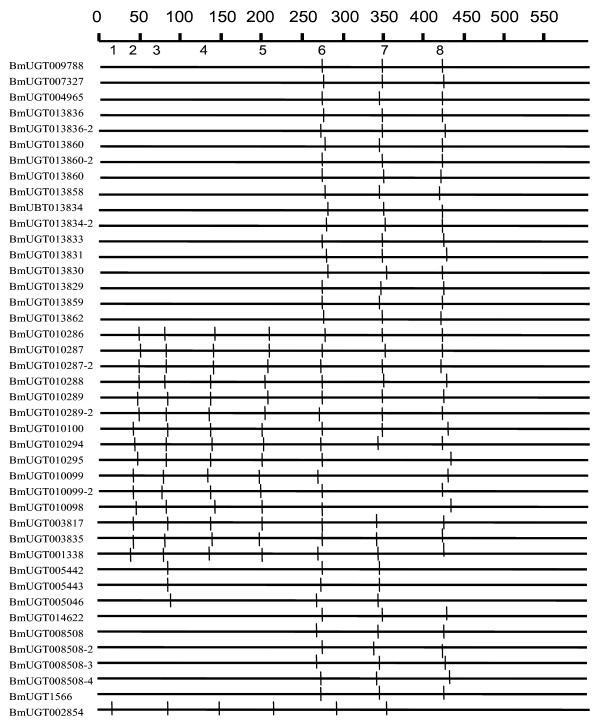
**Distribution of introns among 42 UGTgenes of *B. mori***. The introns are mapped and numbered to the alignment of their amino acid sequences. Black solid lines indicate positions of introns that are found or predicted in the corresponding genes. The numbers on the top of the map show the intron insertion number occurred on each gene.

Phylogenetic tree of silkworm UGTs was reconstructed by the neighbor-joining (NJ) method using a conserved C-terminal region. 5 major groups were defined by this method with high bootstrap supports, we named them groups I-V (Figure 2). Group I contains 12 UGTs; all these genes are tandem arranged on chromosome 7 (Figure 3). Group II contains 3 UGTs; *BmUGT003817 *and *BmUGT003835 *are located on chromosome 24, but genomic position of *BmUGT001338 *is unknown due to the quality of the silkworm genome sequence. Group III also contains 3 genes; *BmUGT005442 *and *BmUGT005443 *are tandem repeated on chromosome 8, while *BmUGT005046 *is located on chromosome 25. Group IV contains 6 UGTs; except for that the genomic locations of *BmUGT014622 *and *BmUGT1566 *are unknown, other 4 genes are located on chromosome 18 (Figure 3). Group V is the largest group that contains 16 genes; 13 of them are tandem arranged on chromosome 28 (Figure 3), and *BmUGT009788*, *BmUGT007327*, and *BmUGT004965 *are located on chromosomes 2, 3 and 25, respectively. Gene *BmUGT002854 *is located on chromosome 10, and it has the special intron position (Figure 4). The phylogenetic analysis also shows that this gene was not clustered with other silkworm UGT genes. Most of members of group I come from the same chromosome, whereas groups II and V are composed of genes located on different chromosomes.

Compared with *D. melanogaster *(33 UGTs), *A. gambiae *(22 UGTs), and *A. mellifera *(12 UGTs), the silkworm UGTs were greatly expanded in number. From the molecular phylogenetic tree of *B. mori*, *D. melanogaster*, *A. gambiae*, *A. mellifera *UGTs (Figure 2) we can see that group C, D, and E, which defined by *D. melanogaster *UGTs, were Diptera-specific classes, and that they were clustered by *D. melanogaster *and *A. gambiae *UGTs only. The silkworm groups III and IV are clustered with *D. melanogaster *groups A and B, respectively, and each cluster also contains *A. gambiae *and *A. mellifera *UGT genes. This indicates that group A and B are common in Lepidoptera, Diptera and Hymenoptera. In group A, *BmUGT005046 *was a probable ortholog of Am15665 because they were phylogenetically closely related (bootstrap value of 95%). *BmUGT005046*, *BmUGT005442*, *BmUGT005443*, and *Am12751*, *Am15163*, *Am12492*, *Am10367 *formed a cluster, supported by a bootstrap value 67% (Figure 2), suggesting that these genes may have a common ancestor. In addition, *BmUGT005442 *and *BmUGT005443 *are tandem repeated on chromosome 8. Thus, *BmUGT005442 *and *BmUGT005443 *might be created through local duplication. The similar phenomenon was also observed in group B. Groups I, II, and V were silkworm-specific classes. They did not form respective clusters with other insect UGTs; genes of each group were tandem repeated on chromosomes. This indicated that most of UGTs might experience lineage-specific expansion in the silkworm.

### Genomic distribution of UGTs in silkworm

39 of the 42 identified silkworm UGT genes were dispersed on 10 chromosomes and 3 genes on unmapped scaffolds. The distinctive feature of the silkworm UGT family is the grouping of genes into clusters with size ranging from 1 to 13 genes per cluster on different chromosomes (Figure 3) (The chromosomes with less than 3 UGTs not shown). The genes in any particular cluster often show a high degree of sequence similarity each other. There are two major gene clusters, located on chromosomes 7 and 28, which contain 12 and 13 genes, respectively. In addition, there are 4 UGT genes located on chromosome 18. Each of chromosomes 8 and 24 contains 2 UGT genes, while each of chromosomes 2, 3, 10, 12, and 25 has one gene.

There are also clear examples of transpositional gene duplications in the silkworm UGT family. For example, *BmUGT005046*, *BmUGT005442*, and *BmUGT005443 *were of the same group III but located on different chromosomes. In the group V, the gene *BmUGT004965*, *BmUGT007327*, and *BmUGT009788 *are respectively located on chromosomes 25, 3, and 2.

### Intron gain and loss as well as intron positions and sizes of silkworm UGTs

Our study revealed that all of 42 silkworm UGTs contained introns. Comparing intron positions with sequence relationships revealed by phylogenetic analysis, about eight independent intron insertion events appear to have happened in the course of the silkworm UGT evolution (Figure 4). The widespread and probably the oldest intron is intron 6, which is found in all of 42 silkworm UGT genes. All other introns are found to be gained or lost only within a single restricted subgroup or in only a single gene. This suggests a general pattern of intron gain during evolution of the UGT gene family. A clear case of one recent intron loss is seen in the group I. Eight genes of this group contain 7 introns, while other four genes have 6 introns. It is likely that the lost one is intron 7, which exists in all other 38 UGTs. This implies that an intron loss event might have occurred after the gene duplication.

In total, at least 180 introns could be identified for the 42 silkworm UGT genes (Figure 4). Each group revealed by phylogenetic analysis almost has the same intron number and the intron positions. Most members of group I have 7 introns with numbers from 2 to 8, while genes *BmUGT010295*, *BmUGT010098*, *BmUGT010099*, and *BmUGT010099-2 *contain 6 introns, which lost intron 7. The members of group II also contain 7 introns and their intron positions are the same as those of the group I. Each gene of group III contains 3 introns and their intron positions are 3, 6, and 7. Each gene of groups IV and V also contains 3 introns, but the intron positions were different from group III; they were 6, 7, and 8 (Figure 4). *BmUGT*002854 contains 6 introns. This gene has the special intron position 1. The silkworm UGTs have more introns compared with *Drosophila *UGTs.

**Figure 5:**
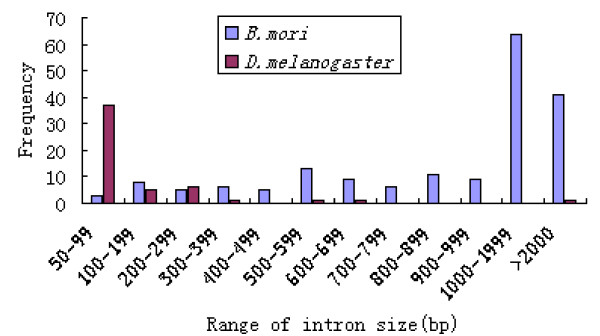
Distribution of the *B. mori *and *D. melanogaster *UGTs intron length.

Intron size of silkworm UGTs ranged from several decades bp to ten thousands bp, and its average was about 1700 bp. About 58.3% of the silkworm UGT introns have sizes > 1000 bp. The silkworm UGTs have longer introns compared with the introns of *D. melanogaster *UGTs (Figure 5), which the majority of introns were 50–99 bp long.

### Expression of silkworm UGT genes

**Figure 6:**
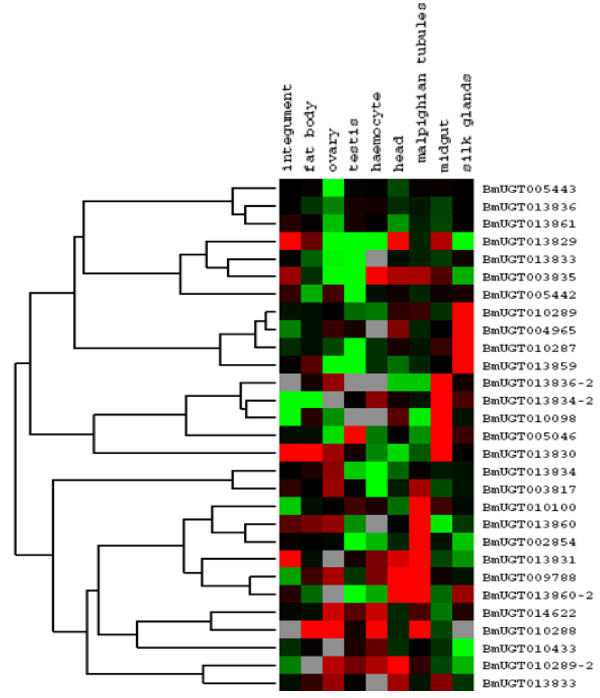
**Expression levels for silkworm UGT genes in different tissues of 3 day 5th larvae by microarray analysis**. Red color represent positive; black color represent zero; green color represent negative; gray color represent missing.

**Figure 7:**
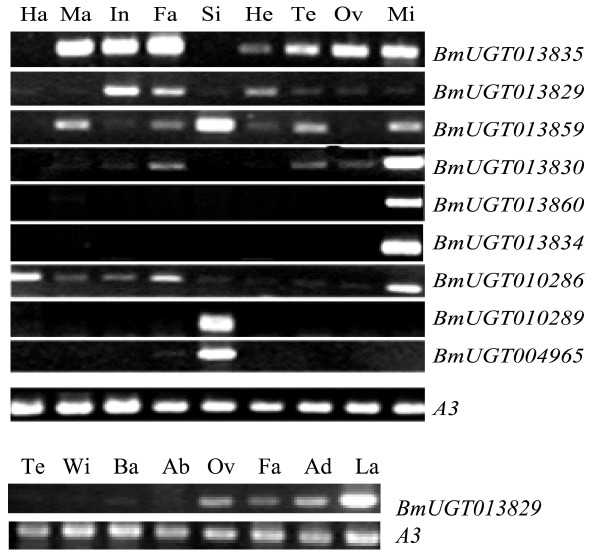
**A Tissue expression patterns of some typical silkworm UGTs in multiple tissues on day 3 of the fifth instar**. RT-PCR amplification of total RNA with *BmUGT*-specific oligonucleotides. Ha, haemocyte; Ma, Malpighian tubules; In, integument, Fa, fat body; Si, silk gland; He, head; Te, testis; Ov, ovary; Mi, midgut. The silkworm cytoplasmic actin A3 gene (Bmactin3; GenBank accession no. U49854) was used as internal control, and denoted by A3. B – Tissue expression patterns of *BmUGT013829 *gene in adult tissues. Te, testis; Wi, wing; Ba, baenosome; Ab, abdomen; Ov, ovary; Fa, fat body; Ad, adult antenna; La, larval antenna.

The expression of the identified silkworm UGT genes was analyzed with known ESTs and microarray data. Of all putative silkworm UGT genes, 29 have expression evidence confirmed by microarray data and 24 have EST evidence. In total, 36 genes were expressed. According to microarray analysis we know that 21 genes have transcribed activity. Among them 16 genes were transcribed in midgut. *BmUGT013829*, *BmUGT014622*, *BmUGT001338*, *BmUGT003817*, and *BmUGT003835 *were widely expressed in silkworm tissues. Some genes were expressed in a tissue-specific pattern, for example, *BmUGT004965 and BmUGT010289 *were specifically expressed in silk gland. *BmUGT013834 *and so on were merely expressed in midgut. While *BmUGT013860-2*, *BmUGT010100 *and *BmUGT002854 *were only expressed in two tissues midgut and malpighian tubules. However, eight genes were not expressed in 3-day-old fifth-instar larvae tissues based on microarray data, such as *BmUGT013833, BmUGT010288 *and *BmUGT005442 *(Figure 6). The RT-PCR was also done to analyze tissue expression patterns of some representative UGT genes on the fifth-instar day 3 larvae. The tissues included testis, ovary, head, integument, fat body, midgut, haemocyte, malpighian tubules and silk glands, which the same with microarray data detected. The results further confirmed these observations (Figure 7A).

## Discussion

Taking advantage of the newly assembled silkworm genome sequence, we identified the 42 putative members of UGT genes, including a reported silkworm UGT gene. The number of UGT genes in silkworm is larger than *D. melanogaster *UGTs, which contain 33 genes [21]. While in plant of *Arabidopsis *there are about 120 UGTs and mammalian UGT gene superfamily currently has 117 members, they are all quite larger number than insects [22].

Both plant and mammalian UGT sequences contained a signature sequence–a UDP-glycosyltransferase signature: (FVA) - (LIVMF) - (TS) - (HQ) - (SGAC) - G - X(2)- (STG) -X(2)- (DE) - X(6) - P - (LIVMFA) - (LIVMFA) - X(2) - P - (LMVFIQ) -X(2)- (DE) - Q (all amino acids that can occur at a given position are listed inside brackets; X indicated any amino acid [11]). It was reported that this motif has been identified in a range of prokaryotic and eukaryotic organisms. There is no exception of insects UGTs. The signature motif is located on C-terminal of the protein sequence.

So far all the identified UGTs comprise two major functional domains [23]. The N-terminal half, believed to be responsible for binding the aglycone, tends to be less conserved than the C-terminal half, which is thought to bind the UDP-sugar. Aglycones bound by UGTs are highly diverse, hence the low amino acid sequence conservation in the N-terminal region between the members of this family. The silkworm UGTs followed the same pattern, with the C-terminal half showing the highest similarity to other UGTs.

The best-characterised UGTs are the mammalian UDP-glucuronosyltransferases, which transfer glucuronic acid to hydrophobic substrates. These enzymes localized in the lumen of the endoplasmic reticulum and are membrane-bound [24]. Mammalian UGTs have two functional motifs are thought to be important for the topology of proteins within the cell. One is the mammalian UGTs contain the N-terminal signal sequence cleaved on cotranslational segregation into the endoplasmic reticulum [25,26]. The other is the putative hydrophobic transmembrane domain located near the carboxyl terminus of the protein, this domain anchors the enzymes to the membrane region [27,25,29]. The major portion of the protein is located in the ER lumen, including the proposed substrate-binding domains and the catalytic site. The silkworm UGTs also have these domains, an N-terminus signal sequence and hydrophobic transmembrane domain either in N-terminal or C-terminal or in both terminals. Thus, these genes are most likely to be anchored in the endoplasmic reticulum. However, no such motif was identified in *Arabidopsis *UGTs [22], supporting that the plant UGTs are cytoplasmic enzymes, different from insect and mammalian UGTs.

42 members of the silkworm UGT gene family scattered on 10 chromosomes. Phylogenetic analysis of these genes defined 5 consistent groups. The genes on each cluster often show a high degree of sequence similarity. This suggests that several gene duplication events took place during the evolution of this family. These duplication events included both tandem events, where the duplicated copies remain adjacent to each other, and transpositional events, where one copy is translocated to a different chromosomal location. Examples of both types of event can be seen in the clade including *BmUGT007327*, *BmUGT002854 *and *BmUGT009788*.

At least 180 introns were identified in 42 silkworm UGTs; the average introns number of each gene is 4.5. However, in *D. melanogaster *each UGT genes have 1.8 introns, quite smaller number than silkworm UGTs; the size of UGTs from the two organisms are similar, all about 500 amino acids. The increase of number of introns in silkworm UGTs is probably due to a fact that the silkworm genome harbors a large proportion of repetitive sequences. And the majority of these repetitive sequences were transposable elements or the remainders of transposable elements. Insertions of transposition into the introns might result in the increase of their lengths.

The micoarray data and RT-PCR results from fifth-instar day 3 larvae tissues showed that the silkworm UGT genes exhibited widely different patterns of expression. Different expression profiles indicate that these UGT genes might have different functions. *BmUGT001338 *and *BmUGT003817 *were expressed in all most tissues and different developmental stages according to microarray data, this indicated that these genes might have important functions in silkworm development and play the housekeeping role. The RT-PCR results showed that *BmUGT010286 *were transcripted in all the tissues, just a low expression was observed in head, silk glands, testis and ovary and possessed similar tissue expression pattern with *BmUGT1*, which reported play a major role in detoxication responses [30]. In addition the two genes located on the same chromosome and may have a common ancestor and phylogenetic tree showed that they were classified into a sub-group. These indicated that *BmUGT010286 *might involved in the detoxication of plant allelochemicals. *B. mori *larvae take up flavonoids into their cocoons from the leaves of their host plant, the mulberry tree (*Morus alba*) [31], but the flavonoids in mulberry tree leaves were different from that which isolated from the cocoon shell of the silkworm [32]. In insects, the formation of glucoside is the predominant pathway for dietary flavonoids [33-36], and the glucosylation of polyphenolics in insects is catalyzed by UDP-glucosyltransferase (UGT) [14,15], *B. mori *UGTs changed the flavonoids glucose conjugation positions from 3-O-glucoside to 5-O-glucoside or other forms to increase fitness for their own. Since a flavonoid 5-O-glucoside has not yet been reported in plants to other animals. Transfer a glucose moiety to the C-5 position of the flavonols, is functioning in *B. mori *[37]. It also was reported that quercetin 5-O-glucoside was the predominant metabolite in the midgut tissue, while quercetin 5,4'-di-O-glucoside was the major constituent in the haemocyte and silk glands [37]. The genes were highly or specific expressed in silk glands, midgut and haemlymph such as *BmUGT010289*, *BmUGT004965*, *BmUGT013859*, *BmUGT003835*, *BmUGT013829*, *BmUGT013830*, *BmUGT013860*, *BmUGT013834*, *BmUGT010286 *might have functions in flavonoids metabolism in silkworm. This may be a very important function for silkworm and further study should be needed to confirm this inference in future. The RT-PCR results also indicted that *BmUGT003835 *gene was highly expressed in testis, ovary, integument, fat body, midgut, malpighian tubules and with lower expression in head, but hardly detectable in haemocyte and silk glands. This expression profile suggested this gene might have some functions in detoxication since the intergument, fat body, and midgut are the main tissues correspond to such activity. We know little about what roles this gene plays in the testis and ovary. It is interesting and worthy of further study. In *D. melanogaster*, there were several UGT genes involved in olfaction; they were preferentially expressed in the third antennal segment of *D. melanogaster *[20]. Both microarray data and RT-PCR indicated that *BmUGT013829 *has high expression levels in silkworm head and RT-PCR also shows that this gene is highly expressed in larval and adult antennae (Figure 7B), suggesting that this gene may be involved in olfaction, but the expression of this gene is not antennae-specific. It can be also detected in fat body and integument, suggesting this gene may have other functions. With more and more insects UGT genes were identified, individual silkworm UGTs functions can initially be determined through bioinformatic studies that reveal homology to genes encoding enzymes of known catalytic activity.

It is known that many plant phenolics can act as toxins or feeding deterrents to insects and thus play an important role in plant defense against herbivorous insects. The detoxication of ingested plant phenolics is believed to be one of the principle functions of insect UGT enzymes [14]. Compared with *A. gambiae*, *D. melanogaster*, and *A. mellifera *UGTs, *B. mori *has more UGTs. Probably this is the result of competition between silkworm and its only diet mulberry leaf. In order to defense *B. mori*, mulberry leaf can produce some toxicant chemicals, while *B. mori *can also evolve some mechanism to detoxicate the chemicals. UGTs probably are involved in this process, so the number of the silkworm UGTs was expanded and also produce the silkworm-specific UGT genes.

## Conclusion

Biochemical evidence and comparisons with mammalian and other systems point to a range of important functions for the UGT genes of this family. Our results indicate that the *B. mori *contains the largest insect UGT gene family characterized to date compared with other insects. The data presented in this study provide an overview for the silkworm UGTs and facilitate their functional studies in future.

## Methods

### Identification of silkworm UGT members

The new version of the silkworm genome sequence and predicted protein database were used in the present analysis . Complete protein sequences of *A. gambiae *and *A. mellifera *were downloaded from Ensemble (AgamP3.45) and BeeBase (release 2), respectively.

UGT protein sequences of *D. melanogaster *were downloaded from the GenBank  and used as queries to perform BLASTP searches against the silkworm predicted protein database and TBLASTN searches against the silkworm 9× genome sequence. A UGT signature motif in a known silkworm UGT gene and *D. melanogaster *UGTs was also used in a TBLASN search. We collected all the candidates if they have UGT signature motif that also exists in plants and mammals. We also used the program SMART to identify whether UDPGT domain exists in the protein sequences encoding by the candidate genes. The same methods were used for identification of the *A. gambiae *and *A. mellifera *UGT genes. Genomic sequences that showed even weak sequence similarity to any query sequence and its flanking regions were extracted, and put into Softberry database for predicting new genes by using FGENESH program taking the available insect (*A. gambiae*, or *D. melanogaster*, or *Tribolium castaneum*, or *A. mellifera*) or the human genome sequence as a reference. For those fragments which their complete coding sequence could not be found by above methods, we used the complete silkworm UGT protein sequences as queries to perform TBLASTN searches against the silkworm 9× genome database and defined the sequence structure by hand.

### Sequence alignment and phylogenetic analysis

Multiple sequence alignments were initially made using the program ClustalX version 1.81 with default gap penalties [38]. These alignments were then reconciled and further adjusted by eye to minimize insertion/deletion events. A conserved C-terminal region about 240–250 amino acids were used in the subsequent phylogenetic analyses, which includes the UGT signature motif. Phylogenetic tree were reconstructed using the neighbor-joining method [39] implemented in MEGA 4.0 program [40]. Bootstrap support was evaluated based on 1000 replicates.

### Gene expression analysis with ESTs and microarray data

More than 184201 ESTs from *B. mori *were available in the National Center for Biotechnology Information (NCBI) database. To search transcriptional evidence for individual UGT genes, a BLASTN search was conducted against the silkworm EST database. The putative coding sequences were used as queries. A 95% or greater identity and minimum cut-off E-value (≤ e-20) were employed to discriminate between duplicated genes. Methods for microarray data analysis were mainly as described in Xia et al. (2007) [41].

### RNA extraction and RT-PCR

Total RNA was extracted both from the fifth-instar day 3 larvae tissues including testes, ovary, head, integument, fat body, midgut, haemocyte, malpighian tubules, silk glands, larval antenna and adult tissues including testis, wing, baenosome, abdomen, ovary, fat body, adult antenna using Trizol reagent (Invitrogen, USA). The concentration of RNA was calculated by a spectrophotometer (Gene Spec V: HITACHI, Japan). DNA within RNA samples were digested with RNase-free DNase I. The first strand of cDNA was synthesized using M-MLV Reverse Transcriptase following the manufacturer's instructions (Promega, USA).

The Primers were designed on the basis of the coding sequences of the silkworm UGTs (see Additional file 1). Silkworm cytoplasmic actin A3 gene (forward primer: 5'-AACACCCCGTCCTGCTCACTG-3'; reverse primer: 5'-GGGCGAGACGTGTGATTTCCT-3') was used as an internal control. PCR amplification was performed in a total reaction volume of 25 μl, containing normalized cDNA, 15 pmol of each primer, 2 mM MgCl_2_, 0.25 mM dNTP, 1× buffer and 2.5 units of Taq DNA polymerase. PCRs were performed with the following cycles: initial denaturation at 94°C for 3 min; then followed by 25 cycles of 30 s at 94°C, 1 min annealing (temperatures listed in Additional file 1), 1.5 min extension (72°C), and a final extension at 72°C for 10 min. The amplification products were analyzed on 1% agarose gels.

## Authors' contributions

FFH carried out the analysis of the sequences and drafted the manuscript. CLC revised the manuscript. ZZ improved the study design and revised the manuscript. ZHL helped to perform the experiment of RT-PCR. FYD revised the manuscript. CL conceived of the study, and participated in its design and coordination and helped to draft the manuscript. ZHX supervised the study. All authors read and approved the final manuscript.

## Supplementary Material

Additional file 1 **Primers and annealing temperature of some silkworm UGT genes**. The data provided represent the primers and annealing temperature of some UGT genes in the silkworm, which were used in the RT-PCR method. Click here for file
