# Supplementary material for: The UDP-glucosyltransferase multigene family in Bombyx mori
Source: BMC Genomics. 2008 Nov 27;9:563. doi: 10.1186/1471-2164-9-563 (PMC2633020; doi:10.1186/1471-2164-9-563)
Supplement: Additional file 1 — Primers and annealing temperature of some silkworm UGT genes. The data provided represent the primers and annealing temperature of some UGT genes in the silkworm, which were used in the RT-PCR method. [file 1471-2164-9-563-S1.doc]

| Gene  name | Forward primer (5'-3') | Reverse primer (5'-3') | Annealing temperature(℃) |
| --- | --- | --- | --- |
| *BmUGT003835* | TACCACGTGCTCTGCGTGT | TCACCTATCATTGCTCAGCAT | 56 |
| *BmUGT013829* | GCAAGGATTTTGGGAATAT | TCAGCTTCCCTTAAACTTAA | 52 |
| *BmUGT013859* | GCTCGGATCCTAGGCGTGT | TCACCTTTCGTCACCAGTGA | 58 |
| *BmUGT013830* | AAGTCATCAAGTAGTCTTCCGA | TTAGTAAATCAGGTTGTGGCAG | 56 |
| *BmUGT013860* | ACTAACGGTAAAGAAGCGGC | GCAGACCACCTTGGGTAATG | 47 |
| *BmUGT013834* | GCCATCAATAAGTCATCAAGTT | AAGCTCCCAAATACACCACAT | 45 |
| *BmUGT010286* | CGTTGAGTGGATGTATTGCGA | AAATACTTGGTTGTGGTGCCC | 56 |
| *BmUGT010289* | TACCCTGTAAAACTGCCGCCC | CGAAGCCCTTCTTTGAAACCA | 60 |
| *BmUGT004965* | GCTCAAGGCGCTTCGATAT | TTACAAAGCCTTGAATTTTCC | 57 |
